# Hierarchically Porous Nitrogen‐Doped Carbon with High Conductivity for Rapid and Efficient Cr(VI) Reduction

**DOI:** 10.1002/advs.202518926

**Published:** 2025-11-28

**Authors:** Danyan Lin, Jie Yang, Fengfeng Chen, Xishi Tai, Zhongshan Chen, Xinrong Guo, Xiangke Wang, Wen Yao

**Affiliations:** ^1^ Dongguan Key Laboratory of Public Health Laboratory Science School of Public Health Guangdong Medical University Dongguan 523808 P. R. China; ^2^ College of Chemistry and Chemical Engineering Weifang University Weifang 261061 P. R. China; ^3^ MOE Key Laboratory of Resources and Environmental Systems Optimization College of Environmental Science and Engineering North China Electric Power University Beijing 102206 P. R. China; ^4^ Lab of Functional Porous Materials School of Materials Science and Engineering Zhejiang Sci‐Tech University Hangzhou 310018 P. R. China

**Keywords:** conductivity, Cr(VI) reduction, hierarchically porous structure, ionic liquid, metal–organic frameworks

## Abstract

Carbon‐based materials derived from metal–organic frameworks typically exhibit microporous structures and low conductivity, which significantly limit their catalytic activity. Herein, an effective strategy to prepare dodecahedral hierarchical porous nitrogen‐doped carbon‐based composites (d‐PNC) by using ZIF‐8 encapsulated with ionic liquid as pyrolysis precursors for efficient Cr(VI) reduction is developed. The encapsulated ionic liquid helps to precisely regulate the hierarchically porous structure in d‐PNC. This hierarchically porous structure not only creates a favorable reaction microenvironment, facilitating the mass transfer of Cr species and their interaction with active sites, but also enhancing the conductivity of d‐PNC and consequently accelerating the electron transfer of •CO_2_^−^ radicals to Cr species, thereby speeding up the reduction process of Cr(VI). Additionally, with the calcination temperature increasing, the content of defective C increases, and N species progressively transforms into graphitic‐center N (N3). Density functional theory calculations reveal that the defective C active center substantially decreases the free energy change of the rate‐determining step (from Cr(IV) to Cr(III)) through the synergistic effect of N3. Given these outstanding characteristics, the optimized d‐PNC material can completely reduce Cr(VI) (333.3 mg g^−1^) in an oxalic acid solution within 2 min, outperforming its counterparts without a hierarchical structure and those calcined at significantly lower temperatures.

## Graphical abstract

The d‐PNC(1100, 10%) catalyst, synthesized via a zinc‐based ionic liquid self‐sacrificing pore‐forming strategy to create a defect‐rich, hierarchical, nitrogen‐doped porous structure, enhances conductivity, promotes rapid mass transfer and synergistic catalytic activity, driving efficient Cr(VI) reduction in OA solution to generate Cr(III)‐OA complexes.

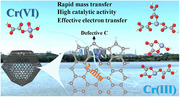

## 1 Introduction

Chromium (Cr), as a toxic heavy metal, is primarily polluted through industrial activities such as metallurgy, electroplating, leather tanning, mining, and pigment production, entering the environment via the discharge of Cr‐containing wastewater.^[^ ^1^, ^2^ ^]^ In aquatic environments, Cr exists in two stable oxidation states: hexavalent (Cr(VI)) and trivalent (Cr(III)).^[^ ^3^ ^]^ Cr(VI) exists as oxyanions (e.g., HCrO_4_^−^, Cr_2_O_7_^2−^) and is characterized by high mobility, solubility, and extreme toxicity, leading to its classification as a Group 1 carcinogen by the World Health Organization. The US Environmental Protection Agency has set the drinking water concentration limit for Cr(VI) at 0.05 mg L^−1^.^[^ ^4^, ^5^, ^6^ ^]^ In contrast, Cr(III) is less toxic and can be readily removed through precipitation. Therefore, reducing the more hazardous Cr(VI) to Cr(III) is a widely applicable strategy for Cr(VI) removal. Recently, the use of small‐molecule organic acids for Cr(VI) reduction has garnered much attention. However, the catalytic performance of a single small molecule organic acid is limited, and a synergistic high‐performance catalyst is needed to achieve remarkable results and meet the requirements for high‐performance treatment of Cr‐containing wastewater.^[^ ^7^ ^]^

**Figure 1:**
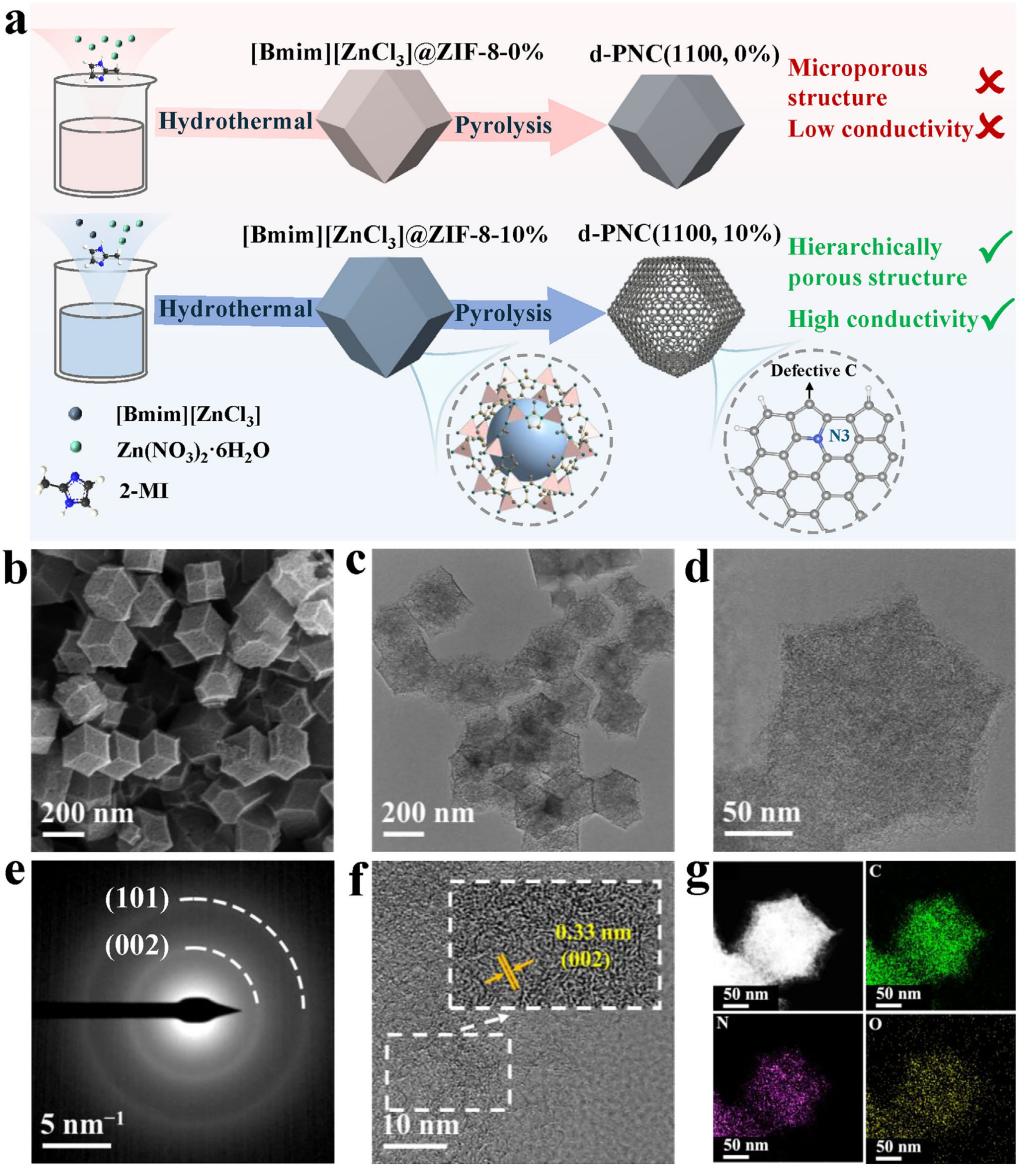
a) Schematic illustration of the synthesis of d‐PNC(1100, 0%) and d‐PNC(1100, 10%). b) SEM image, c,d) TEM images, e) SAED pattern, f) HRTEM image with lattice distortions, and g) HAADF‐STEM image and corresponding EDX elemental mapping images of d‐PNC(1100, 10%).

Recently, carbon‐based materials derived from metal–organic frameworks have gained widespread attention in catalysis, energy conversion, adsorption and separation, and other fields due to their high porosity, large specific surface area, and excellent stability.^[^ ^8^, ^9^, ^10^, ^11^ ^]^ Among these, the nitrogen‐rich zeolite imidazole framework‐8 (ZIF‐8) is considered as an ideal precursor for synthesizing nitrogen‐doped porous carbon materials.^[^ ^12^, ^13^ ^]^ However, carbon‐based materials derived from pure ZIF‐8 typically exhibit only microporous structures, which hinder the mass transfer of Cr species and result in a significant number of active sites being buried, thereby limiting their catalytic activity.^[^ ^14^, ^15^, ^16^, ^17^, ^18^, ^19^ ^]^ While the template method is generally effective for generating larger pore sizes, the hard template approach often involves etching steps with strong acids or bases, leading to hazardous waste and complicating the manufacturing process.^[^ ^20^, ^21^ ^]^ Conversely, the soft template method can clog pores due to residual templates, ultimately resulting in a lower specific surface area and reduced exposure of active sites.^[^ ^22^, ^23^ ^]^ Furthermore, for catalytic reactions involving electron transfer, such as the reduction of Cr(VI) to Cr(III), enhancing the conductivity of carbon materials is crucial for improving catalytic performance. Unfortunately, carbon‐based materials derived from pure ZIF‐8 generally exhibit limited conductivity in addition to defects in pore structure (**Figure** **1**a). Given these challenges, developing an effective strategy to improve the pore structure (mesopores or macropores) and conductivity in ZIF‐8‐derived carbon materials is of great significance for enhancing the reduction performance of Cr(VI).

Herein, we developed a strategy to prepare a novel dodecahedral hierarchical porous nitrogen‐doped carbon‐based catalyst (d‐PNC) through controlled pyrolysis of ZIF‐8 encapsulated with ionic liquid (IL) under an inert atmosphere. During the pyrolysis process, the introduce of IL created a hierarchical pore structure with enhanced conductivity in d‐PNC material, which synergistically improved the catalytic performance of the material in Cr(VI) reduction. The resulting d‐PNC catalyst achieved complete reduction of Cr(VI) in an oxalic acid (OA) solution within 2 min, significantly higher than that of the microporous carbon material derived from pure ZIF‐8. We demonstrate that this improvement is due to its excellent characteristics, which provide rapid mass transport capability, abundant accessible active sites, and ultrafast electron transfer. We believe that this innovative strategy not only opens a new avenue for designing materials for Cr(VI) removal but also provides a novel method for constructing carbon materials with a hierarchically porous structure and high conductivity.

## 2 Results and Discussion

The synthetic procedures for d‐PNC(1100, 10%) were illustrated in Figure 1a. The Zinc‐based IL ([Bmim][ZnCl_3_]) (Figure S1, Supporting Information) and Zn(NO_3_)_2_·6H_2_O were co‐dissolved in a methanol solution at a specific ratio (m(Zn(NO_3_)_2_·6H_2_O:m([Bmim][ZnCl_3_]) = 10:1, 10%). Meanwhile, the 2‐methylimidazole (2‐MI) ligand was separately dispersed in another portion of methanol. The metal source solution was gradually added to the ligand solution. During this process, ZIF‐8 crystallized and encapsulated the IL in situ through its micropores, forming the composite precursor [Bmim][ZnCl_3_]@ZIF‐8‐10% (Figure S2, Supporting Information). Subsequently, the precursor was calcined under flowing nitrogen at 1100 °C for 2 h. At high temperature, the ZIF‐8 skeleton carbonizes into a porous nitrogen‐doped carbon matrix. This process resulted in a hierarchically porous nitrogen‐doped carbon‐based composite material d‐PNC(1100, 10%).

Scanning electron microscopy (SEM) image and transmission electron microscopy (TEM) images revealed that the [Bmim][ZnCl_3_]@ZIF‐8‐10% material exhibited a dodecahedral morphology, which was consistent with traditional ZIF‐8 materials (Figures S3–S5, Supporting Information), indicating that encapsulating the IL did not alter the original morphology of ZIF‐8. The powder X‐ray diffraction (XRD) pattern demonstrated that [Bmim][ZnCl_3_]@ZIF‐8‐10% still maintained high crystallinity as that of traditional ZIF‐8 materials (Figure S6, Supporting Information).^[^ ^24^ ^]^ To confirm the encapsulation of IL in ZIF‐8, we conducted Fourier transform infrared (FT‐IR) and N_2_ adsorption–desorption tests. The FT‐IR spectra showed that the emergence of a new vibration peak (≈877 cm^−1^) in the [Bmim][ZnCl_3_]@ZIF‐8‐10%, corresponding to the Zn─Cl bond in the IL, suggesting that the IL may be encapsulated in the pores of ZIF‐8 (Figure S7, Supporting Information).^[^ ^25^ ^]^ The N_2_ adsorption–desorption results indicated a decrease in specific surface area and pore volume of the [Bmim][ZnCl_3_]@ZIF‐8–10% material compared to pure ZIF‐8 without encapsulating the IL, confirming that the [Bmim][ZnCl_3_] IL was successfully encapsulated in the pores of ZIF‐8 (Figure S8, Supporting Information).

After pyrolysis, the SEM image (Figure 1b) and TEM images (Figure 1c,d) revealed that the as‐prepared d‐PNC(1100, 10%) retained the initial dodecahedral shape of the starting polyhedral (Figures S3,S5, Supporting Information). The bright rings in the selected‐area electron diffraction (SAED) patterns, featuring (002) and (101) diffraction rings confirmed the polycrystalline graphite structure (Figure 1e). The high‐resolution TEM (HRTEM) image clearly showed 0.33 nm lattice fringes corresponding to the graphite (002) planes (Figure 1f). Furthermore, the high‐angle annular dark‐field scanning TEM (HAADF‐STEM) image and energy‐dispersive X‐ray spectroscopy (EDX) elemental mapping images conclusively demonstrated the well‐preserved dodecahedral architecture while confirming the homogeneous distribution of C, N, and O elements within the porous framework (Figure 1g).

**Figure 2:**
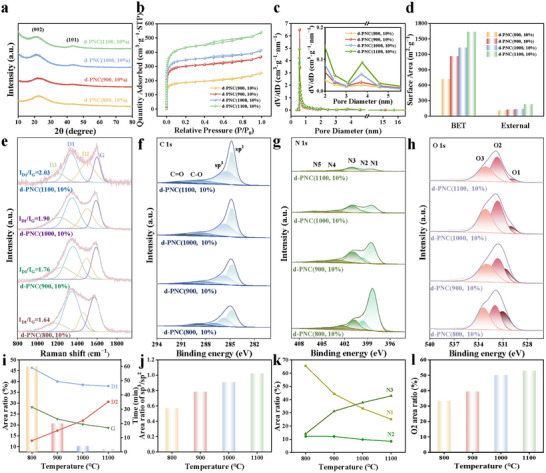
The structural properties of d‐PNC(800, 10%), d‐PNC(900, 10%), d‐PNC(1000, 10%), and d‐PNC(1100, 10%), synthesized under different temperature regimes, were investigated: a) XRD patterns, b) N_2_ adsorption–desorption isotherms, c) pore‐size distributions, d) histograms of BET and external surface areas, e) Raman spectra, high resolution XPS spectra of f) C 1s, g) N 1s, h) O 1s, i) Raman peaks area and reaction completion time, j) the corresponding sp^3^/sp^2^ C ratios in C 1s, k) peaks area of N species in N 1s, and l) O2 peak area in O 1s.

The XRD patterns of d‐PNC(1100, 10%) revealed characteristic graphite diffraction peaks associated with the (002) and (101) crystallographic planes (**Figure** **2**a). The morphology of the (101) diffraction peak reflected the degree of structural order of graphite microcrystals in the carbon matrix.^[^ ^26^ ^]^ The Raman spectra of d‐PNC(1100, 10%) exhibited characteristic peaks at 1347 cm^−1^ (D band) and 1594 cm^−1^ (G band), designated as D band related to the characteristic of sp^3^ defect and G band related to sp^2^‐boned graphitic carbon, respectively (Figure 2e).^[^ ^27^ ^]^ Defect‐related peaks emerged at 1211 cm^−1^ (D3 band) and 1500 cm^−1^ (D2 band). The D2 band originated from nitrogen‐doped carbon defects within graphitic layers, while the D3 band was associated with structural distortions in non‐planar carbon networks.^[^ ^27^ ^]^ The *I*_D1_/*I*_G_ ratio of 2.03 and the D2‐type defect proportion of 30.67% indicated enriched nitrogen‐doped carbon edge defects (Figure 2i). The surface chemical states of the d‐PNC(1100, 10%) were probed by X‐ray photoelectron spectroscopy (XPS) (Figure S9, Table S2, Supporting Information). In Figure 2f, the high‐resolution C 1s spectra can be deconvoluted into C sp^2^, C sp^3^, C─O, and C═O.^[^ ^27^, ^28^ ^]^ Notably, the C sp^3^/sp^2^ peak area ratio, a measure of defect density, was 1.021 for d‐PNC(1100, 10%) (Figure 2j). The N 1s spectra in Figure 2g were deconvoluted into five nitrogen configurations including pyridinic N (N1), pyrrolic N (N2), graphitic‐center N (N3), graphitic‐valley N (N4), and oxidized N (N5).^[^ ^29^ ^]^ The O 1s spectra resolved into three distinct components: lattice oxygen (O3), defective oxygen (O2), and carbonate species (O1) (Figure 2h).^[^ ^30^ ^]^ In d‐PNC(1100, 10%), oxygen vacancy‐related states (O2) accounted for 52.93% (Figure 2l). N_2_ adsorption–desorption analysis of d‐PNC(1100, 10%) revealed a combined type I/IV isotherm profile (Figure 2b, Table S1, Supporting Information). At low relative pressures, the adsorption capacity exhibited progressive enhancement with increasing pressure. A distinct hysteresis loop emerged within the 0.4–0.9 *P*/*P*_0_ range, indicative of hierarchical micro‐mesoporous architecture. Pore size distribution analysis confirmed the coexistence of micropores (<2 nm) and mesopores (2–50 nm) within the material (Figure 2c). The BET surface area measured 1639.90 m^2^ g^−1^, with an external surface area of 230.43 m^2^ g^−1^ (Figure 2d). Total pore volume reached 0.83 cm^3^ g^−1^ (Table S1, Supporting Information).

**Figure 3:**
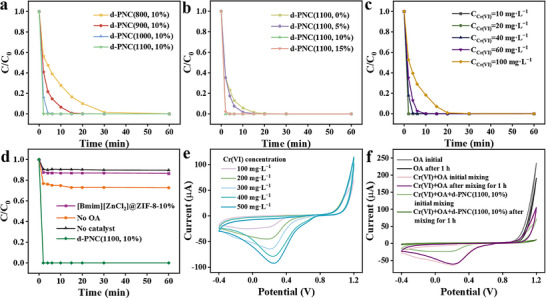
Effects of a) pyrolysis temperature, b) IL doping amount, c) initial Cr(VI) concentration, and d) reaction system composition. CV curves in different e) Cr(VI) concentrations and f) reaction system compositions. (Reaction parameters: catalyst dosage = 0.06 g L^−1^, C_Cr(VI)_initial = 20 mg L^−1^, C_OA_ = 0.24 g L^−1^, room temperature, pH_initial_ = 2.5 without addition extra acid. No catalyst = Cr(VI) + OA, No OA = d‐PNC(1100, 10%) + Cr(VI), different catalysts = catalysts + Cr(VI) + OA).

Next, d‐PNC(1100, 10%) was utilized as a catalyst for Cr(VI) reduction. Research showed that when combined with OA, d‐PNC(1100, 10%) demonstrated efficient catalytic performance, achieving full reduction of Cr(VI) in just 2 min, with a removal performance of 333.3 mg g^−1^, which was much rapider and higher than most of the catalysts in previous studies (**Figure** **3**a,b, Table S3, Supporting Information). In addition, both low (10 mg L^−1^) and high (100 mg L^−1^) concentrations of Cr(VI) can be completely removed within 2 and 30 min by d‐PNC(1100, 10%), respectively (Figure 3c). In contrast, using pure OA or d‐PNC(1100, 10%) alone resulted in limited removal rates of only 9.5% and 23.3%, respectively (Figure 3d). The reliability of using cyclic voltammetry (CV) to measure Cr(VI) concentration was confirmed by the linear relationship between reduction peak current and concentration (Figure 3e; Figure S10, Supporting Information). A one‐hour co‐exposure with OA significantly reduced the Cr(VI) reduction peak current (Figure 3f), providing direct electrochemical evidence for high Cr(VI) removal efficiency and supporting the catalytic performance trends from Figure 3d, which demonstrated the enhancement of the overall Cr(VI) reduction ability of the d‐PNC(1100, 10%) by OA. This highlighted that the effective removal of Cr(VI) was due to the synergistic interaction between OA and d‐PNC(1100, 10%). While multiple low‐molecular‐weight organic acids contributed to Cr(VI) reduction,^[^ ^31^, ^32^ ^]^ research utilizing d‐PNC(1100, 10%) catalysis has demonstrated OA as the superior option for this purpose (Figure S11, Supporting Information), which was attributed to its unique molecular structure that enabled the synergy of efficient reduction and anti‐passivation. Its unstable C─C bond ensured rapid reaction kinetics.^[^ ^33^ ^]^ It also formed soluble complexes with the generated Cr(III) to prevent the coating of the catalyst by Cr(III) precipitation and maintained the catalytic efficiency.^[^ ^34^ ^]^ We also found that the dosage of OA and the amount of catalyst significantly influence the removal performance of Cr(VI). The optimal molar ratio of Cr(VI) to OA is 1:7, with an ideal catalyst dosage of 0.06 mg L^−1^ (Figures S12,S13, Supporting Information). The efficiency of Cr(VI) reduction at this OA ratio appeared linked to the favorable OA speciation and proton availability at the resulting pH.^[^ ^35^ ^]^ Furthermore, the catalytic performance of the [Bmim][ZnCl_3_]@ZIF‐8‐10% precursor was substantially lower than that of the calcined d‐PNC(1100, 10%), underscoring the importance of high‐temperature pyrolysis. Additionally, varying calcination temperatures can greatly impact the catalytic performance of the materials.

To investigate the effect of temperature, a series of other carbon materials by adjusting the calcination temperature (800–1000 °C), named as d‐PNC(800, 10%), d‐PNC(900, 10%), and d‐PNC(1000, 10%), respectively, were prepared. SEM images indicated that the three d‐PNC materials displayed the same dodecahedral morphology as d‐PNC(1100, 10%) (Figure S14, Supporting Information). XRD patterns of the three d‐PNC materials showed characteristic graphite diffraction peaks similar to those of d‐PNC(1100, 10%) (Figure 2a). With the increase of pyrolysis temperature from 800 to 1100 °C, the structure of the catalyst changed from amorphous to ordered graphite. Correspondingly, the diffraction peak of (101) developed from a broad peak that was difficult to identify to a clear peak shape, indicating that the order degree of graphite microcrystals continued to increase.^[^ ^26^ ^]^ Raman spectroscopy analysis revealed a progressive increase in the *I*_D1_/*I*_G_ ratio (d‐PNC(800, 10%): 1.64; d‐PNC(900, 10%): 1.76; d‐PNC(1000, 10%): 1.90), confirming that high‐temperature treatment significantly enhanced the defect density of the carbon skeleton (Figure 2e). Concurrently, the proportion of D2‐type defects also increased with temperature, reflecting the enrichment of defects (Figure 2i). XPS analysis further demonstrated that the sp^3^/sp^2^ peak area ratio in the C 1s spectra increased from 0.569 (d‐PNC(800, 10%)) to 1.021 (d‐PNC(1100, 10%)), indicating elevated complexity of defects under high‐temperature conditions (Figure 2f,j). The N 1s spectra showed that as the pyrolysis temperature increased, the ratio of unstable N1 and N2 decreased significantly while stable N3 and N4 gradually became the dominated doping structures. Importantly, as the pyrolysis temperature increased to 1100 °C, we observed a notable increase in the proportion of N3 from 14.18% to 42.80% (Figure 2g,k). This indicated that high temperatures were more inclined to form a thermodynamically more stable N3 structure. Additionally, the O 1s spectra revealed an increase in defect‐related oxygen species O2 from 33.53% to 52.93%, attributed to the enrichment of oxygen vacancies and low‐coordination oxygen species, which optimized charge transfer pathways (Figure 2h,l). As illustrated in Figure 2b,c, the N_2_ adsorption/desorption isotherms of the catalysts exhibited more pronounced hysteresis loops with increasing temperature, suggesting the formation of additional mesoporous structures during calcination. The BET and external surface areas increased progressively with temperature increase, while the average pore volume expanded from 0.39 cm^3^ g^−1^ in d‐PNC(800, 10%) to 0.83 cm^3^ g^−1^ in d‐PNC(1100, 10%) (Figure 2d, Table S1, Supporting Information).

The d‐PNC(800, 10%), d‐PNC(900, 10%), and d‐PNC(1000, 10%) were also used for Cr(VI) reduction. The results indicated that the catalytic reduction performance of Cr(VI) on these carbon materials including d‐PNC(1100, 10%) exhibited significant temperature dependence. As the calcination temperature increased, the catalytic activity of the materials gradually improved, with the d‐PNC(1100, 10%) sample demonstrating optimal performance (**Figure** **3**a). Based on the above characterization analysis results, we reasonably speculated that the high catalytic activity of d‐PNC(1100, 10%) was attributed to the high defect carbon content, possibly as well as the synergistic effect of graphite nitrogen sites at different positions. On the other hand, the enlarged pore structure, combined with abundant exposed active sites, optimized mass transport pathways during the reaction, thereby enhancing the catalytic performance.

**Figure 4:**
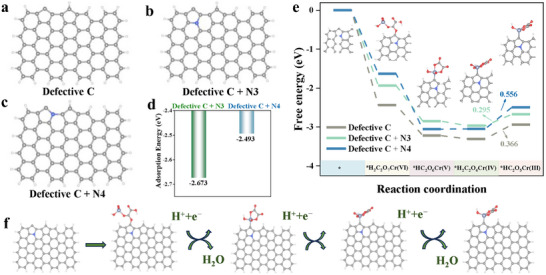
Modeling of a) Defective C, b) Defective C + N3, and c) Defective C + N4 active sites. d) Adsorption energies on Defective C + N3 and Defective C + N4. e) Gibbs free energy diagram depicting the reduction reaction of Cr(VI) and OA on Defective C, Defective C + N3, and Defective C + N4. f) Model construction showing the interaction between OA and Cr(VI) in Defective C + N3.

The influence of the structural evolution described above on the intrinsic catalytic activity of active sites remained to be thoroughly explored. The prior studies had confirmed Defective C as the central active site in nitrogen‐doped carbon materials and demonstrated that synergistic interactions between defective carbon and nitrogen could enhance catalytic performance.^[^ ^36^, ^37^ ^]^ In this study, calcination at 1100 °C induced significant increases in both defect concentration and graphitic nitrogen content (N3 and N4 configurations) within the catalyst. These observations collectively implied a potential thermal reordering of priority among nitrogen species as active sites. To clarify this mechanism, density functional theory (DFT) simulations were performed by constructing models of Defective C, Defective C + N3, and Defective C + N4 embedded, utilizing structural parameters derived from experimental characterizations (Figure S15, Table S4, Supporting Information, **Figure** **4**). The adsorption energies of Cr(VI)‐OA on Defective C + N3 and Defective C + N4 were −2.673 and −2.493 eV, respectively (Figure 4d). The negative adsorption energies indicated that the reactions were exothermic, and the absolute value of the adsorption energy suggested that the interactions between Cr(VI)‐OA and the catalyst was stronger with larger values. The adsorption energy of Cr(VI) on Defective C + N3 is greater than that on Defective C + N4, suggesting that Defective C + N3 may play a more significant role in the initial adsorption of Cr(VI). To further identify which configuration of graphitic nitrogen acts as the primary active site in the OA‐mediated Cr(VI) reduction, we examined the free energy profiles illustrated in Figure 4e. The rate‐determining step (RDS), defined by the highest free energy change (*ΔG*), was the electron transfer step. For the Defective C, Defective C + N3, and Defective C + N4 catalysts, the RDS was the reduction step from *H_2_C_2_O_6_Cr(IV) to *HC_2_O_5_Cr(III), with corresponding activation energies of 0.366, 0.295, and 0.556 eV (Figure 4e,f). The lowest RDS energy on Defective C + N3 indicated its superior catalytic activity. Both the adsorption energy calculations and the *ΔG* calculations consistently pointed to a stronger interaction between Cr(VI) and the Defective C + N3 sites, which confirmed that Defective C + N3 were indeed the thermodynamically favored active centers for the reduction of Cr(VI) to Cr(III).

The above DFT simulations elucidated the electronic synergistic mechanism between N3 and Defective C. However, the actual catalytic efficiency of the material often depended on whether reactants could efficiently reach the active sites—a process highly reliant on the hierarchical nature of the pore structure and the spatial distribution of active sites. The TGA curves (Figure S16, Supporting Information) of the IL‐doped sample showed a distinct weight loss at ≈766 °C, matching ZnCl_2_ volatilization and absent in the pure ZIF‐8 ([Bmim][ZnCl_3_]@ZIF‐8‐0%). The gas released from IL decomposition contributed to the formation of a hierarchical pore structure in the carbon matrix. Therefore, we investigated the influence of [Bmim][ZnCl_3_] doping amount on the structure and properties of d‐PNC. Similarly, the as‐synthesized materials were denoted as d‐PNC(1100, 0%), d‐PNC(1100, 5%), d‐PNC(1100, 10%), and d‐PNC(1100, 15%), respectively, corresponding to the different [Bmim][ZnCl_3_]/Zn^2+^ mass ratios. The [Bmim][ZnCl_3_]@ZIF‐8‐x materials were initially prepared by introducing varying masses of [Bmim][ZnCl_3_] into Zn(NO_3_)_2_ solution. Apparently, the morphology and crystalline lattice structure of [Bmim][ZnCl_3_]@ZIF‐8‐x were consistent with ZIF‐8 (Figures S3,S6, Supporting Information). The nitrogen adsorption isotherm of [Bmim][ZnCl_3_]@ZIF‐8‐x (Figure S8, Supporting Information) exhibited typical type I microporous isotherm characteristics, and its specific surface area decreased with the increase of the amount of IL [Bmim][ZnCl_3_], which was attributed to the introduction of [Bmim][ZnCl_3_]. In addition, the average pore volume of ZIF‐8 was significantly reduced (Table S1, Supporting Information), indicating that [Bmim][ZnCl_3_] was encapsulated in the pores of ZIF‐8.

**Figure 5:**
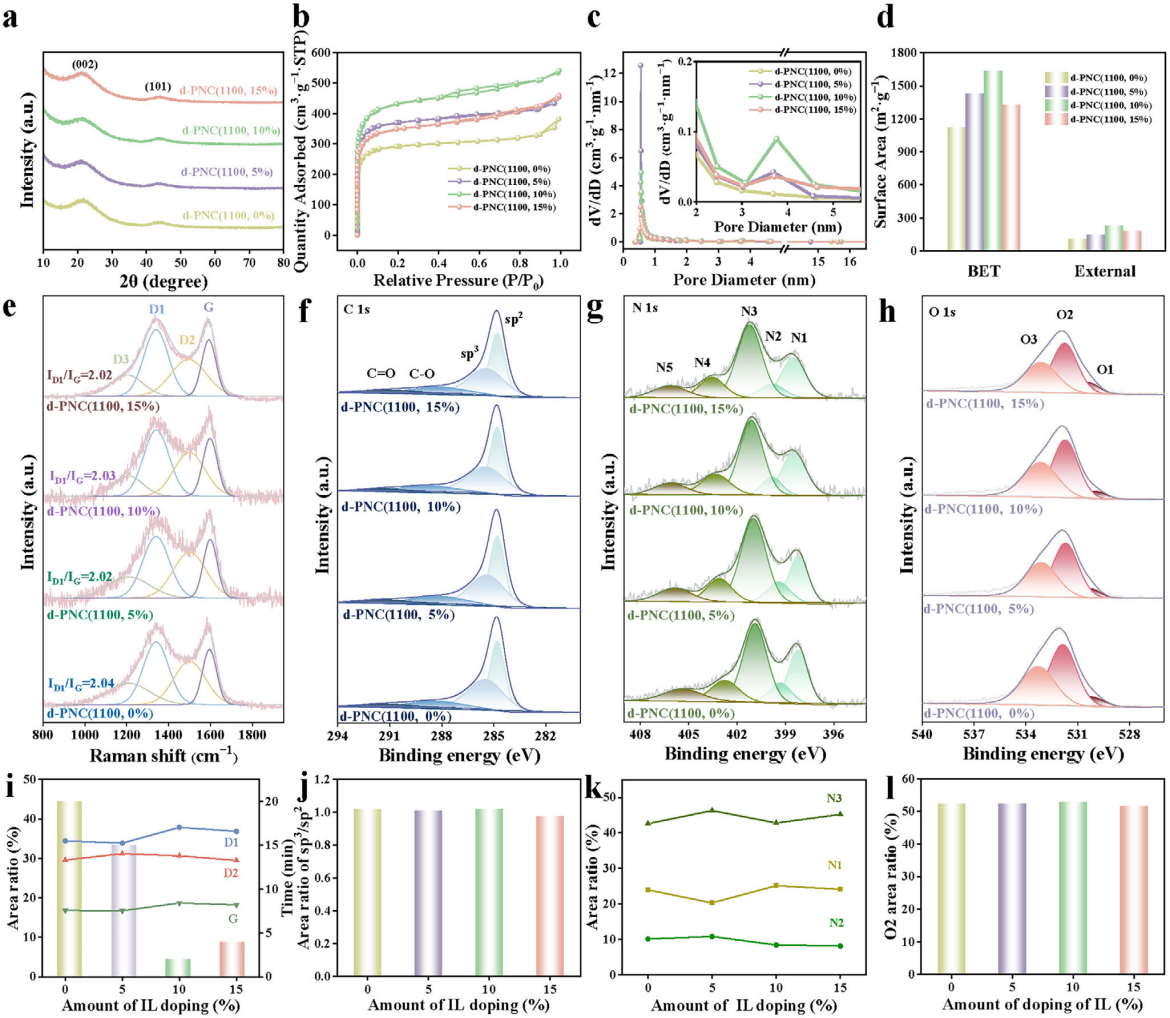
The structural properties of d‐PNC(1100, 0%), d‐PNC(1100, 5%), d‐PNC(1100, 10%), and d‐PNC(1100, 15%), synthesized under different amount of IL doping, were investigated: a) XRD patterns, b) N_2_ adsorption–desorption isotherms, c) pore‐size distributions, d) histograms of BET and external surface areas, e) Raman spectra, high resolution XPS spectra of f) C 1s, g) N 1s, h) O 1s, i) Raman peaks area and reaction completion time, j) the corresponding sp^3^/sp^2^ C ratios in C 1s, k) peaks area of nitrogen species in N 1s, and l) O2 peak area in O 1s.

Subsequently, the precursor was pyrolyzed at 1100 °C to obtain the corresponding carbon materials. It was observed that the addition of different IL did not have a significant effect on the morphology and lattice structure of the carbon catalysts (**Figure** **5**a; Figures S17,S18, Supporting Information). However, varying the doping concentration influenced catalyst performance, and a concentration of 10% resulted in the greatest Cr(VI) reduction (Figure 3b). The loading of [Bmim][ZnCl_3_] had a significant threshold control effect on the pore structure and properties of ZIF‐8‐derived materials, with the critical value being ≈10%. Specifically, increasing the loading from 0% to 10% resulted in an increase in the BET surface area from 1123.78 to 1636.90 m^2^ g^−1^, accompanied by an increase in external surface area from 108.04 to 230.43 m^2^ g^−1^, as well as a relative increase in mesopore content (Figure 5b–d). These findings indicated that appropriate doping facilitated the development of a mesoporous structure via evaporation etching. However, exceeding the 10% loading threshold led to a decrease in both BET surface area (to 1326.30 m^2^ g^−1^) and external surface area (to 180.59 m^2^ g^−1^), along with a decline in mesopore content, suggesting that excessive doping can induce pore collapse. Crucially, Raman and XPS analyses revealed that the extent of doping did not alter the inherent defect level and N3 content of the material (Figure 5e–l; Figure S19, Supporting Information), confirming that changes in catalytic activity were primarily mediated by the presence, stability, and accessibility of the mesoporous structure. These results further suggested that an appropriate amount of the IL dopant favored the formation of an accessible, interconnected mesoporous network, leading to enhanced exposure of active sites.

To reveal the reduction products of the Cr(VI) reduction system, we conducted a series of characterizations on the materials and products following the reaction. EDX elemental mapping images revealed the presence of Cr alongside C, N, and O, homogeneously distributed across the d‐PNC(1100, 10%) material (Figure S20, Supporting Information). The Cr 2p spectrum displayed peaks at 586.75 and 577.36 eV, characteristic of Cr(III) species (Figure S21, Supporting Information), indicating successful reduction of Cr(VI).^[^ ^38^, ^39^ ^]^ While C 1s spectra showed unchanged sp^2^, sp^3^, C─O, and C═O bonds, shifts in the N 1s and the O 1s spectra, suggested Cr(III) coordination to N and O functionalities (Figure S22, Supporting Information). UV–vis spectroscopy of the post‐reaction solution (Figure S23, Supporting Information) confirmed complete Cr(VI) reduction by the disappearance of its characteristic peak at ≈350 nm. The presence of absorption peaks at ≈426 and ≈590 nm, similar to those of Cr(III), confirmed Cr(VI) reduction to Cr(III). A blue shift in the Cr(III) peaks relative to pure CrCl_3_ solution indicated the formation of complexes between Cr(III) and OA.^[^ ^40^ ^]^ Macroscopic changes also supported Cr(VI) reduction. The original paleyellow solution became colorless (Figure S24, Supporting Information), visually indicating the removal of Cr(VI). The subsequent addition of CaO and CaCl_2_ quickly generated a precipitation, which, upon drying, was confirmed to be CaCr_2_O_4_ (PDF# 48–0203) and Cr_2_O_3_ (PDF# 38–1479) via XRD, exhibiting the characteristic reflections of that crystalline phase. Collectively, these lines of evidence demonstrated the efficacy of d‐PNC(1100, 10%) in promoting Cr(VI) reduction to Cr(III).

To directly confirm the presence of Cr(V) intermediates, we conducted time‐resolved electron paramagnetic resonance (EPR) measurements. The chelating agents 2‐ethyl‐2‐hydroxy butanoic acid (EHBA) for Cr(IV) and Cr(V) were introduced into each reaction system. We captured the characteristic peak belonging to Cr(V). Special attention should be paid to analyzing the characteristic EPR spectral changes of the Cr(V)‐EHBA complex in the Cr(VI)/EHBA system. The EPR signal of Cr(V) gradually weakened, indicating that Cr(V) did not accumulate but further transformed into a low‐valence state of Cr species (Figure S25, Supporting Information). At the same time, no EPR signal of the Cr(IV)‐EHBA complex was detected, indicating that Cr(V) was rapidly reduced to Cr(III).

**Figure 6:**
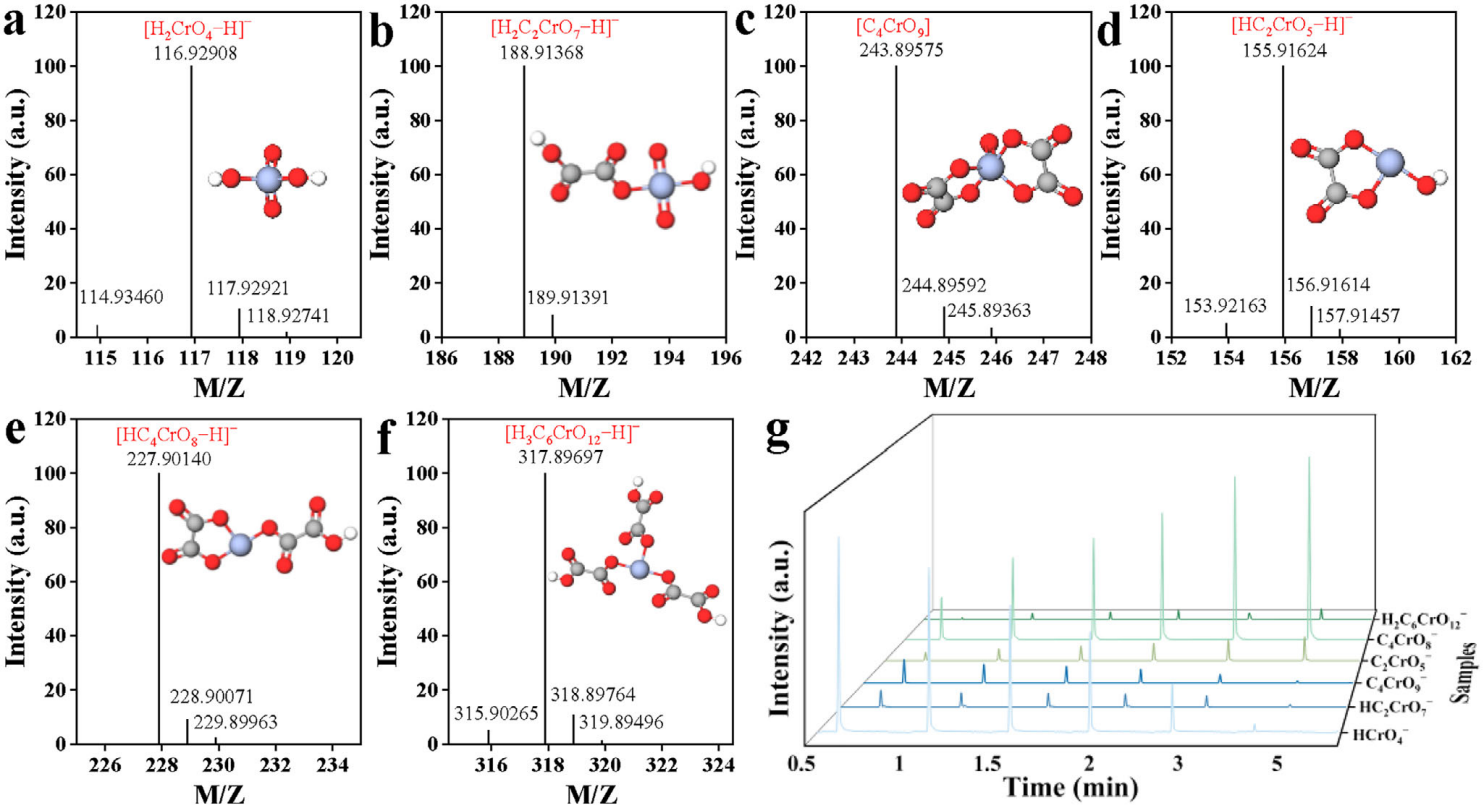
The HRMS spectrum of Cr(VI) reduction intermediates in OA solution: a) HCrO_4_^−^, b) HC_2_CrO_7_^−^, c) C_4_CrO_9_, d) C_2_CrO_5_^−^, e) C_4_CrO_8_^−^, and f) H_2_C_6_CrO_12_^−^. g) Concentration profiles of Cr intermediates during reduction measured by HRMS.

To further identify the intermediates and final products formed during the process, high‐resolution mass spectrometry (HRMS) was employed to capture Cr‐OA complexes in the reaction solution. OA functioned as an electron donor, supplying electrons required for Cr(VI) reduction reactions. Furthermore, it coordinated with chromate anions to form complexes. The results indicated that six Cr‐containing species (**Figure** **6**a–f, Table S5, Supporting Information) were present in the reaction system, which were respectively assigned to Cr(VI) intermediates [HCrO_4_^−^, m/z = 116.92908; HC_2_CrO_7_^−^, m/z = 188.91368], a Cr(V) intermediate [C_4_CrO_9_, m/z = 243.89575], and Cr(III) products [C_2_CrO_5_^−^, m/z = 155.91624; C_4_CrO_8_^−^, m/z = 227.90140; H_2_C_6_CrO_12_^−^, m/z = 317.89697]. The results of HRMS indicated a significant reduction in the peak intensities associated with Cr(VI)‐OA and Cr(V)‐OA complexes during the reaction, accompanied by a corresponding increase in the peak intensity of the Cr(III)‐OA complex (Figure 6g). The HRMS analysis provided strong support for the reduction of Cr(VI) to Cr(III) by OA. While identifying intermediate Cr‐OA complexes with varying oxidation states, the detection and characterization of Cr(III)‐OA clearly demonstrated the formation of Cr(III) as the terminal product of the Cr(VI) reduction process.

**Figure 7:**
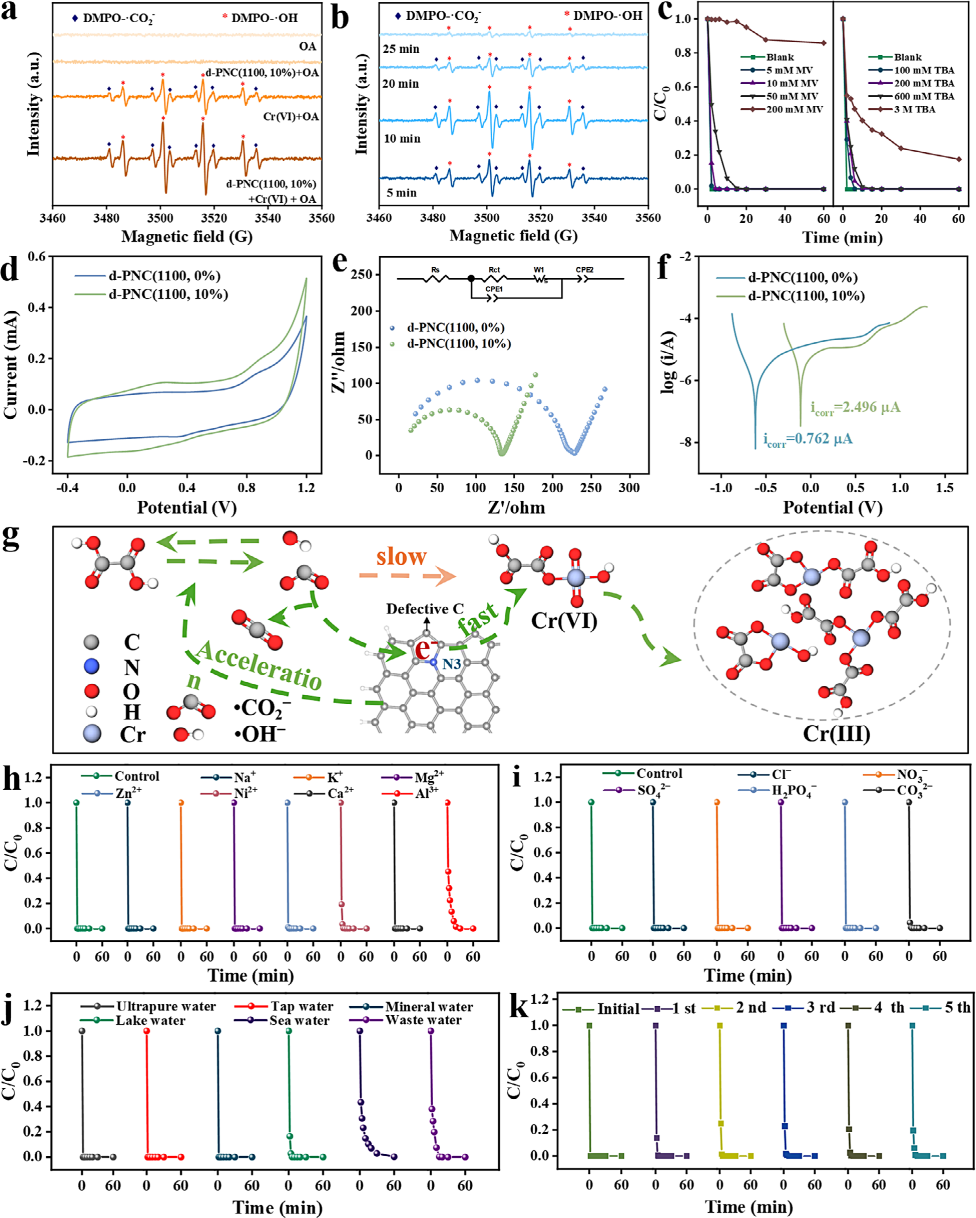
DMPO capture EPR profiles of •OH and •CO_2_^−^ in different a) reaction systems and b) time. c) Effect of different radical bursters on d‐PNC(1100, 10%)‐mediated reduction of Cr(VI) by OA. Electrochemical characterization of d) CV curves, e) EIS and f) Tafel polarization curves. g) Proposed Cr(VI) removal mechanism of d‐PNC(1100, 10%) coupling with OA. Effects of h) coexisting cations, i) coexisting anions, and j) actual water matrix on Cr(VI) reduction efficiency. k) Cyclic stability of d‐PNC(1100, 10%) in Cr(VI) reduction with OA. (Performance experiment reaction parameters: catalyst dosage = 0.06 g L^−1^, C_Cr(VI)_initial = 20 mg L^−1^, C_OA_ = 0.24 g L^−1^, ion concentration = 1 mM, room temperature, pH_initial_ = 2.5 without the addition extra acid).

Previous studies had found that OA will decompose and produce •OH and •CO_2_^−^ radicals, which can reduce Cr(VI).^[^ ^41^ ^]^ To unravel the reaction mechanisms of free radicals, EPR spectroscopy was employed with 5,5‐dimethyl‐1‐pyrroline N‐oxide (DMPO) as a spin‐trapping agent to detect •OH and •CO_2_^−^ radicals under simulated visible‐light irradiation (xenon lamp) (**Figure** **7**a,b).^[^ ^42^ ^]^ No EPR signals were detected in the OA solution by itself and d‐PNC(1100, 10%)/OA coexisted solution after 10 min of irradiation, indicating minimal radical generation from OA photolysis under these conditions. Distinct DMPO‐•OH (1:2:2:1 quartet) and DMPO‐•CO_2_^−^ signals emerged exclusively in the Cr(VI)/OA system (Figure 7a),^[^ ^43^ ^]^ with signal intensity amplified upon introducing the d‐PNC(1100, 10%) catalyst, validating its role in promoting OA‐derived radical generation. When d‐PNC(1100, 10%)/Cr(VI)/OA coexisted for 20–25 min, weak or no signals were detected (Figure 7b), likely due to reactions between radicals and Cr(VI), possibly forming intermediate Cr species and complexes. Quenching experiments using tert‐butanol (•OH scavenger) and methyl viologen (•CO_2_^−^ scavenger) further established the mechanistic dominance of these radicals. Dose‐dependent inhibition of Cr(VI) removal efficiency was observed for both scavengers, with higher concentrations leading to more pronounced declines in reduction rate and efficiency (Figure 7c). These results unequivocally demonstrated the critical roles of •OH and •CO_2_^−^ radicals in mediating Cr(VI) reduction.

The coexistence of OA and Cr(VI) can generate active free radicals. However, the reduction performance of Cr(VI) was significantly improved in the presence of an added catalyst. We speculated that the catalyst may possess an additional capability, such as facilitating electron transfer. To investigate this, electrochemical testing was conducted to evaluate the performance of the catalysts related to electron transfer in redox reactions. Specifically, CV curves (Figure 7d) revealed that d‐PNC(1100, 10%) exhibited enhanced redox activity due to its higher surface area and abundant redox‐active sites, resulting in a high specific capacitance indicative of improved electron buffering and robust electron transfer within the material.^[^ ^44^ ^]^ Supporting these findings, electrochemical impedance spectroscopy (EIS) revealed a significantly lower charge transfer resistance at the electrode–electrolyte interface (Figure 7e; Figure S26, Table S6, Supporting Information), implying faster electron transfer kinetics.^[^ ^45^ ^]^ Tafel polarization (Figure 7f) further indicated that d‐PNC(1100, 10%) presented a greater density of active sites, as evidenced by a higher corrosion current (Icorr = 2.496 µA). This result suggested an enhanced electron transfer from •CO_2_^−^ to Cr(VI), thereby enhancing the reduction process.^[^ ^46^ ^]^ The transient photocurrent test (Figure S27, Supporting Information), demonstrated the highly efficient separation efficiency of photogenerated carriers of d‐PNC(1100, 10%). Collectively, d‐PNC acted as an electron transfer medium through its conductive network to construct an efficient directional electron transport path from •CO_2_^−^ to Cr(VI) through the carbon framework. Effectively overcame the traditional homogeneous system of mass transfer limitations (Figure 7g).

Evaluating the potential for practical application was a crucial aspect of materials development. The anti‐interference capability was crucial in evaluating the practical feasibility of catalysts and reduction technologies. The influence of several cations (i.e., Na^+^, K^+^, Zn^2+^, Mg^2+^, Ni^2+^, Ca^2+^, and Al^3+^) on the reduction of Cr(VI) by d‐PNC(1100, 10%) was assessed, and the results are presented in Figure 7h. Introducing Na^+^, K^+^, Zn^2+^, Mg^2+^, and Ca^2+^ had a negligible impact on Cr(VI) reduction efficiency. While Ni^2+^ and Al^3+^ exhibited a discernible influence, potentially due to chelation with OA, d‐PNC(1100, 10%) nevertheless achieved complete Cr(VI) removal within 30 min. The impact of coexisting anions on the catalytic process was depicted in Figure 7i. Common anions (H_2_PO_4_^−^, NO_3_^−^, SO_4_^2−^, Cl^−^, and CO_3_^2−^) demonstrated minimal interference with Cr(VI) reduction. Collectively, these findings suggested that d‐PNC(1100, 10%) exhibited robust anti‐interference characteristics within this reaction system.

Following the assessment of d‐PNC(1100, 10%)’s tolerance to common interfering ions, its performance in removing Cr(VI) from various relevant aqueous matrices was evaluated (Figure 7j). Initially, to exert precise control over Cr(VI) concentrations and to elucidate the influence of differing water compositions, Cr(VI) reduction experiments were conducted using tap water, lake water, mineral water, and seawater fortified with Cr(VI) (Table S7, Supporting Information). The Cr(VI) concentration in these water samples was adjusted to 20 mg L^−1^. Across these systems, the reduction capacity of d‐PNC(1100, 10%) toward Cr(VI) remained largely consistent, with the exception of seawater. Specifically, in the seawater system, a minor attenuation in Cr(VI) reduction efficiency of d‐PNC(1100, 10%) was observed, potentially attributable to its inherent complexity and elevated ionic strength. Subsequent to demonstrating effective Cr(VI) removal from fortified natural water samples, the performance of d‐PNC(1100, 10%) was assessed using a bona fide industrial wastewater sample containing Cr(VI). For this purpose, it was tested on electroplating wastewater, characterized by an initial Cr(VI) concentration of 107 mg L^−1^. When the reaction time was extended to 30 min, all Cr(VI) in the wastewater can also be completely reduced. These findings underscored the environmental relevance and potential for addressing Cr(VI) contamination in both diverse water sources and industrial settings. Furthermore, by increasing d‐PNC(1100, 10%) loading to four times the initial amount, the material demonstrated effective U(VI) (10 mg L^−1^) reduction, achieving 97.69% removal in 1 h. Even with the increased loading, this highlighted its potential as a promising catalyst for the simultaneous removal of multiple heavy metal pollutants from water (Figure S28, Supporting Information).

To further assess the long‐term viability and economic feasibility of d‐PNC(1100, 10%), cyclic regeneration experiments were performed. Using a HCl solution (0.1 M) as the desorbent, d‐PNC(1100, 10%) showed remarkable stability, maintaining 100% Cr(VI) removal within 10 min over five cycles (Figure 7k). Used d‐PNC(1100, 10%) exhibited dodecahedral morphology similar to d‐PNC(1100, 10%), confirmed by TEM images (Figure S20a,b,e Supporting Information). The SAED pattern (Figure S20c, Supporting Information) displayed bright rings, and HRTEM (Figure S20d, Supporting Information) showed lattice planes of 0.33 nm, comparable to d‐PNC(1100, 10%), indicating retained crystallinity. EDX elemental mapping images confirmed that C, N, and O were still uniformly distributed throughout the carbon framework (Figure S20f, Supporting Information). These results fully suggested that d‐PNC(1100, 10%) had high stability and reusability under this reaction conditions.

**Figure 8:**
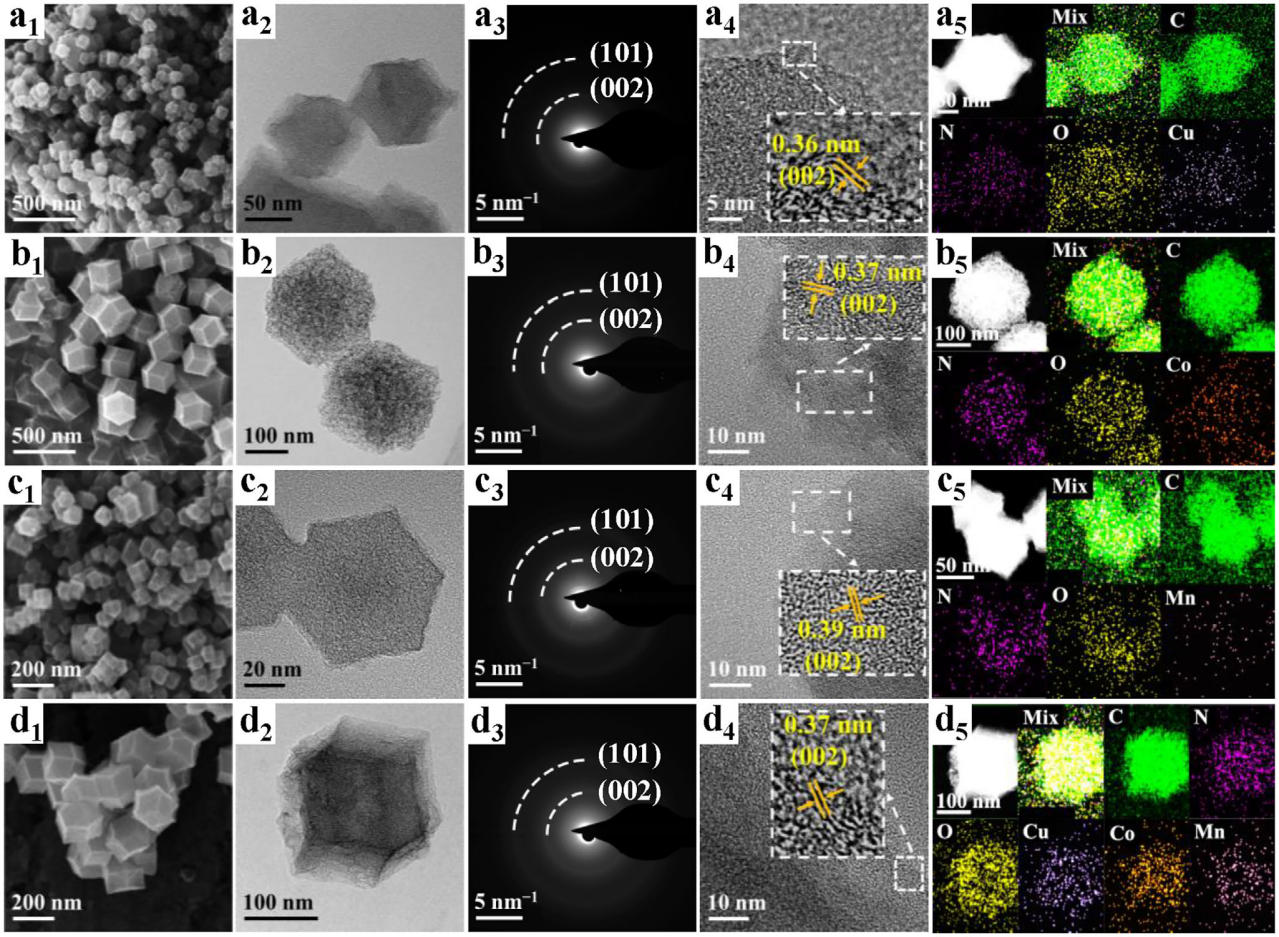
SEM images, TEM images, SAED patterns, HRTEM images with lattice distortions, and HAADF‐STEM images and corresponding EDX elemental mapping images of a_1_–a_5_) Cu/d‐PNC, b_1_–b_5_) Co/d‐PNC, c_1_–c_5_) Mn/d‐PNC, and d_1_–d_5_) Cu‐Co‐Mn/d‐PNC.

The successful fabrication of d‐PNC(1100, 10%), characterized by its tailored properties and exceptional activity, not only established an effective approach for Cr remediation but also provided a robust and adaptable synthetic framework for the rational design and construction of diverse advanced functional materials. The inherent generality of this approach stemmed from the modular design of our synthetic platform. Notably, the core d‐PNC framework demonstrated a remarkable capacity to incorporate various transition metal active sites through precise modulation of the ILs employed during synthesis (**Figure** **8**; Figures S1,S2,S29,S30, Supporting Information). Preliminary investigations revealed that catalysts comprising alternative metals (e.g., Cu/d‐PNC, Co/d‐PNC, Mn/d‐PNC, Cu‐Co‐Mn/d‐PNC), synthesized via this identical methodology, also exhibited significant catalytic activity and maintained elevated levels of efficiency in Cr(VI) reduction, frequently achieving rapid removal kinetics comparable to those observed with d‐PNC(1100, 10%) (Figure S31, Supporting Information). This highly adaptable synthetic strategy facilitated the targeted synthesis of a new generation of metal‐containing catalysts with tunable active centers, thereby expanding the potential to address a broader spectrum of environmental pollutants and enabling exploration within diverse catalytic applications beyond Cr(VI) remediation. The ability to maintain high activity even with different metal centers underscored the robustness and generalizability of the synthesis approach.

## 3 Conclusion

In summary, we developed a pore‐forming strategy using a volatile Zn‐based IL as a self‐sacrificing agent. By leveraging the synergistic effects of the ZIF‐8 microporous framework and IL mesoporous templating, we constructed hierarchically porous nitrogen‐doped carbon materials with a high density of defective carbon and graphitic nitrogen active sites through the high‐temperature volatilization of endogenous IL components. Catalytic activity increased with both temperature and IL doping level (≤10%), as these factors collectively enhanced pore volume, defect density, and conductivity, and improved performance. Experimental results and DFT calculations revealed that the Defective C and N3 active sites of d‐PNC(1100, 10%) synergistically drove OA to generate •CO_2_^−^ radicals, which completely reduced highly toxic Cr(VI) to less toxic Cr(III) within 2 min. Furthermore, the catalyst maintained high catalytic activity after five cycles and showed exceptional effectiveness in removing Cr(VI) from various aqueous environments. Additionally, this synthesis strategy proved to be highly versatile, allowing for the precise construction of various metal–carbon composite systems. Carbon‐based catalysts derived from metal‐containing IL consistently exhibited superior activity, confirming the approach's multifunctionality and establishing a methodological foundation for developing novel high‐performance catalytic materials.

## Conflict of Interest

The authors declare no conflict of interest.

## Supporting information



Supporting Information

## Data Availability

The data that support the findings of this study are available from the corresponding author upon reasonable request.
