# Supplementary material for: Hierarchically Porous Nitrogen‐Doped Carbon with High Conductivity for Rapid and Efficient Cr(VI) Reduction
Source: Adv Sci (Weinh). 2025 Nov 28;13(8):e18926. doi: 10.1002/advs.202518926 (PMC12884740; doi:10.1002/advs.202518926)
Supplement: Supplementary file 1 — Supporting Information [file ADVS-13-e18926-s001.docx]

Supporting Information

**Hierarchically Porous Nitrogen-Doped Carbon with High Conductivity for Rapid and Efficient Cr(VI) Reduction**

*Danyan Lin, Jie Yang, Fengfeng Chen, Zhongshan Chen*, Xinrong Guo*, Xiangke Wang*, Wen Yao**

**Experimental Section**

**Text S1. Materials and agent**

All chemicals (analytical grade) in this work received without further purification. 2-methylimidazole (C_4_H_6_N_2_), diphenyl carbamide (C_13_H_14_N_4_O), zinc nitrate hexahydrate (Zn(NO_3_)_2_·6H_2_O), and o-Phthalic anhydride (C_8_H_4_O_3_) were obtained from Aladdin chemical reagent Co., Ltd (Shanghai, China). 1-butyl-3-methylimidazolium chloride (BmimCl) was purchased from Langzhou greenchem ILs. Potassium chloride (KCl), magnesium chloride (MgCl_2_), nickel (II) chloride hexahydrate (NiCl_2_·6H_2_O), sodium chloride (NaCl), calcium chloride (CaCl_2_), zinc chloride (ZnCl_2_), aluminum chloride (AlCl_3_), phosphoric acid (H_3_PO_4_), sodium hydroxide (NaOH), methyl viologen dichloride (C_12_H_14_Cl_2_N_2_·xH_2_O), copric chloride dihydrate (CuCl_2_·2H_2_O), and manganese chloride tetrahydrate (MnCl_2_·4H_2_O) were purchased from Shanghai Macklin Biochemical Co., Ltd. Oxalic acid dihydrate (H_2_C_2_O_4_·2H_2_O), formic acid (CH_2_O_2_), citric acid (C_6_H_8_O_7_), malonic acid (C_3_H_4_O_4_), tartaric acid (C_4_H_6_O_6_), and tert-Butanol (C_4_H_10_O) were purchased from Shanghai Energy Chemical Co., Ltd. Potassium dichromate (K_2_Cr_2_O_7_), potassium dihydrogen phosphate (KH_2_PO_4_), sodium nitrate (NaNO_3_), sodium carbonate (Na_2_CO_3_) and sodium sulfate (Na_2_SO_4_), were obtained via Guangzhou Chemical Reagent Factory Co., Ltd.

**Text S2. Synthesis**

**Synthesis of [Bmim][ZnCl_3_]**

[Bmim][ZnCl_3_] was synthesized by mixing the equimolar ratio of ZnCl_2_ and BmimCl. Typically, anhydrous ZnCl_2_ (1.36 g, 10 mmol) was added into a round-bottom flask containing BmimCl (1.75 g, 10 mmol) and further stirred under nitrogen atmosphere. The products were heated to 120 °C in a three-necked flask with a reflux condenser for 2 h under stirring and nitrogen atmosphere. The as-prepared ionic liquids were kept in a desiccator.

**Synthesis of [Bmim][CuCl_3_] (M = Cu, Co, and Mn)**

Taking [Bmim][CuCl_3_] as an example, [Bmim][CuCl_3_] was synthesized by mixing the equimolar ratio of CuCl_2_·2H_2_O and BmimCl. Typically, CuCl_2_·2H_2_O (1.70 g, 10 mmol) and BmimCl (1.75 g, 10 mmol) were mixed and further stirred at room temperature until the clear, homogeneous, and transparent liquids appear. The obtained mixture was dried overnight at 100 °C to eliminate the impurities.

The synthesis steps and amounts of relevant reagents used of [Bmim][CoCl_3_] and [Bmim][MnCl_3_] were identical to that of [Bmim][CuCl_3_], except that the metal salt were CoCl_2_ 6H_2_O and MnCl_2_·4H_2_O, respectively.

**Synthesis of [Bmim][****ZnCl_3_]@ZIF-8-x (x = 0%, 5%, 10% and 15%)**

Taking [Bmim][ZnCl_3_]@ZIF-8-10% as a representative example, 2.044 g of 2-MI was dissolved in 22.75 mL of methanol with stirring (15 min, flask A). In parallel, 1.851 g of Zn(NO_3_)_2_·6H_2_O and 0.185 g of [Bmim][ZnCl_3_] were dissolved in 47.25 mL of methanol (15 min stirring, flask B). The solution from flask B was then rapidly added to flask A under vigorous stirring, and the mixture was stirred for 60 min at 30 °C. The resulting solution was transferred to 100 mL Teflon-lined autoclaves and heated to 120 °C at 1 °C·min^−1^ for 4 h. After cooling, the product was isolated by centrifugation (8000 rpm, 3 min), washed three times with methanol, and dried at 80 °C for 12 h. The resulting powder was labeled as [Bmim][ZnCl_3_]@ZIF-8-x, where x represents the mass ratio of [Bmim][ZnCl_3_] to Zn^2+^. For [Bmim][ZnCl_3_]@ZIF-8-0% (x = 0%), [Bmim][ZnCl_3_] were not used. For [Bmim][ZnCl_3_]@ZIF-8-5% (x = 5%), 0.0925 g of [Bmim][ZnCl_3_] were used. For [Bmim][ZnCl_3_]@ZIF-8-15% (x = 15%), 0.2775 g of [Bmim][ZnCl_3_] were used.

**Synthesis of** **[Bmim][MCl_3_]****@ZIF-8 (M = Cu, Co, Mn and** **Cu/Co/Mn)**

Taking [Bmim][CuCl_3_]@ZIF-8 as an example, 2.044 g of 2-MI was dissolved in 22.75 mL of methanol with stirring (15 min, flask A). In parallel, 1.851 g of Zn(NO_3_)_2_·6H_2_O and 0.185 g of [Bmim][CuCl_3_] were dissolved in 47.25 mL of methanol (15 min stirring, flask B). The solution from flask B was then rapidly added to flask A under vigorous stirring, and the mixture was stirred for 60 min at 30 °C. The resulting solution was transferred to 100 mL Teflon-lined autoclaves and heated to 120 °C at 1 °C·min^−1^ for 4 h. After cooling, the product was isolated by centrifugation (8000 rpm, 3 min), washed three times with methanol, and dried at 80 °C for 12 h.

The synthesis steps and amounts of relevant reagents used of [Bmim][CoCl_3_]@ZIF-8 and [Bmim][MnCl_3_]@ZIF-8 were identical to that of [Bmim][CuCl_3_], except that the ionic liquid were [Bmim][CoCl_3_] and [Bmim][MnCl_3_], respectively. For [Bmim][Cu/Co/MnCl_3_]@ZIF-8, 0.0555 g of [Bmim][CuCl_3_], 0.0555 g of [Bmim][CoCl_3_], and 0.074 g of [Bmim][MnCl_3_] were used.

**Synthesis of** **d-PNC(T, A) (T = 800 ℃, 900 ℃, 1000 ℃, and 1100 ℃; A = 0%, 5%, 10%, and 15%) and** **M/d-PNC (M = Cu, Co, and Mn)**

The synthesized [Bmim][ZnCl_3_]@ZIF-8, or [Bmim][MCl_3_]@ZIF-8 was subjected to pyrolysis in a tube furnace. The sample was heated to a target temperature (T) at 2 °C·min^−1^, held at T for 2 h under flowing N_2_, and then cooled to room temperature. The resulting powders, denoted as d-PNC(T, A), and M/d-PNC, were collected. The catalysts were used directly without further processing.

**Text S3. Materials Characterizations**

Scanning electron microscopy (SEM) images were obtained by using Hitachi SU-8000 microscope with an acceleration voltage of 5 kV. Transmission electron microscopy (TEM) images, selected-area electron diﬀraction (SAED) patterns, high-resolution TEM (HRTEM) images, high-angle annular dark-ﬁeld scanning TEM (HAADF-STEM) images and energy-dispersive X-ray spectroscopy (EDX) elemental mapping images were captured on a FEI-Talos F200S instrument microscope. Powder X-ray diffraction (XRD) was measured using a D8 Advanced diffractometer (BRUKER). The scanning rate of XRD is 2°·min^−1^. Raman spectra were measured using a HORIBA JY LabRAM HR Evolution. FT-IR spectra were measured using a Nicolet iS50. The specific surface areas, microporous surface area, and pore size distribution were calculated based on the Brunauer-Emmett-Teller method, t-plot method, and the nonlocal density functional theory method using a Micromeritics ASAP 2460. The element states were investigated by operating X-ray photoelectron spectroscopy (XPS) using a Thermo Fisher Scientific K-Alpha. The thermal stability of the precursor was studied by using thermogravimetric analysis (TG 209 F3 Tarsus) with a N_2_ atmosphere protection and a heating rate of 10 °C·min^−1^.

**Text S4. Performance of dodecahedral hierarchical porous nitrogen-doped carbon-based composite material (d-PNC) for Cr(VI) removal**

To prepare a 50 mL Cr(VI) solution at a concentration of 20 mg·L^−1^, potassium dichromate (K_2_Cr_2_O_7_) was dissolved in deionized water. The intermittent removal experiments of Cr(VI) were conducted in a beaker under magnetic stirring. The concentration of Cr(VI) in the reaction solution was determined using the 1,5-diphenylcarbazide spectrophotometric method (GB 7467-87). The reduction of Cr(VI) mediated by d-PNC was performed at room temperature (30 ± 5 ℃) and atmospheric pressure. A 50 mL potassium dichromate solution was treated with 3.0 mg of catalyst and 17.0 mg of oxalic acid, and the removal of Cr(VI) was monitored. Control experiments using different catalysts or no catalyst were also carried out under identical conditions. At the end of each experiment, the catalyst was collected, washed, and dried for further use and analysis.

The equilibrium removal capacity (*q*_e_, mg∙g^−1^) of Cr(Ⅵ) is calculated based on formula (1).

$q_{e}=\frac{\left( C_{0}-C_{e} \right)}{m}V$ (1)

Here, *C*_0_ and *C*_e_ represented the initial concentration of Cr(Ⅵ) and the concentration of Cr(Ⅵ) in the solution at equilibrium, respectively (mg∙L^−1^); *V* was the volume of the Cr(Ⅵ) solution (L); and *m* was the mass of the material (g).

The 🞄OH produced by OA decomposition can react with the carboxyl group in oxalic acid to form a reducing free radical •CO_2_^−^ (Eq. (2)):

•OH + HC_2_O_4_^−^ → •CO_2_^−^ + CO_2_ + H_2_O (2)

•CO_2_^−^ can transfer electrons through electron shuttle to finally reduce Cr(VI) (Eq. (3)(4)):

•CO_2_^−^ → CO_2_ + e^−^ (3)

Cr(VI) + e^−^ → Cr(V), Cr(V) + e^−^ → Cr(IV), Cr(IV) + e^−^ → Cr(III) (4)

**Text S5. EPR test conditions**

For EPR measurements, concentrations of Cr(VI), and OA, were increased compared to batch experiments to enhance signal intensity and ensure reliable detection. The EPR tests were conducted under the following conditions: catalyst, 0.06 g·L^−1^; Cr(VI), 100 mg·L^−1^; OA, 1.19 g·L^−1^; EHBA, 1000mg/L.

**Text. S6. Electrochemical measurements**

The electrochemical measurements, with a three-electrode cell, were conducted on an electrochemical workstation (CHI660E, Shanghai Chenhua Instrument Co, China) at room temperature. The Hg/HgCl_2_ electrode was selected as the reference electrode; a platinum wire was used as a counter electrode. The working electrode was prepared: 3 mg of catalysts, and 1.2 mL of ultrapure water were mixed by ultrasonic dispersion. Next, 5 μL of the sample suspension was pipetted onto the working electrode and dried. The electrolyte was composed of 0.24 g·L^−1^ OA, and 20 mg·L^−1^ Cr(VI). Cyclic voltammetry (CV) measurement was performed as the potential varied from −0.4 to 1.2 V vs. Hg/HgCl_2_ with a scan rate of 50 mV·s^−1^. Electrochemical impedance spectra (EIS) were recorded at open circuit potential vs. Hg/HgCl_2_ with a frequency range from 10^5^ to 10^−2^ Hz, utilizing an AC voltage at 5 mV amplitude. Tafel polarization profiles were obtained at open circuit potential vs. Hg/HgCl_2_ (VOCP±0.25 V) with a scan rate of 10 mV·s^−1^.

**Text S7. Precipitation process details**

After the reaction was completed, the solution system was chosen to be treated with CaCl_2_ and CaO, as this combination can precipitate Cr(III) and oxalate from the system, achieving a harmless process. Experimental conditions: CaCl_2_ concentration, 0.65 g·L^−1^; CaO concentration, 0.85 g·L^−1^.

**Text S8. Computational method**

Density functional theory (DFT) calculations were performed using the Vienna Ab initio Simulation Package (VASP).^[1,2]^ The generalized gradient approximation (GGA) with the Perdew-Burke-Ernzerhof (PBE) functional was employed.^[3]^ Projected augmented wave (PAW)^[4,5]^ potentials described ionic cores, with valence electrons represented by a plane wave basis set (kinetic energy cutoff: 450 eV). Van der Waals interactions were accounted for using the DFT-D3 correction. Geometry optimizations converged when forces were below 0.02 eV·Å^−1^ and energy changes less than 1 × 10^−5^ eV. A 1×1×1 Gamma-centered k-point mesh was used.


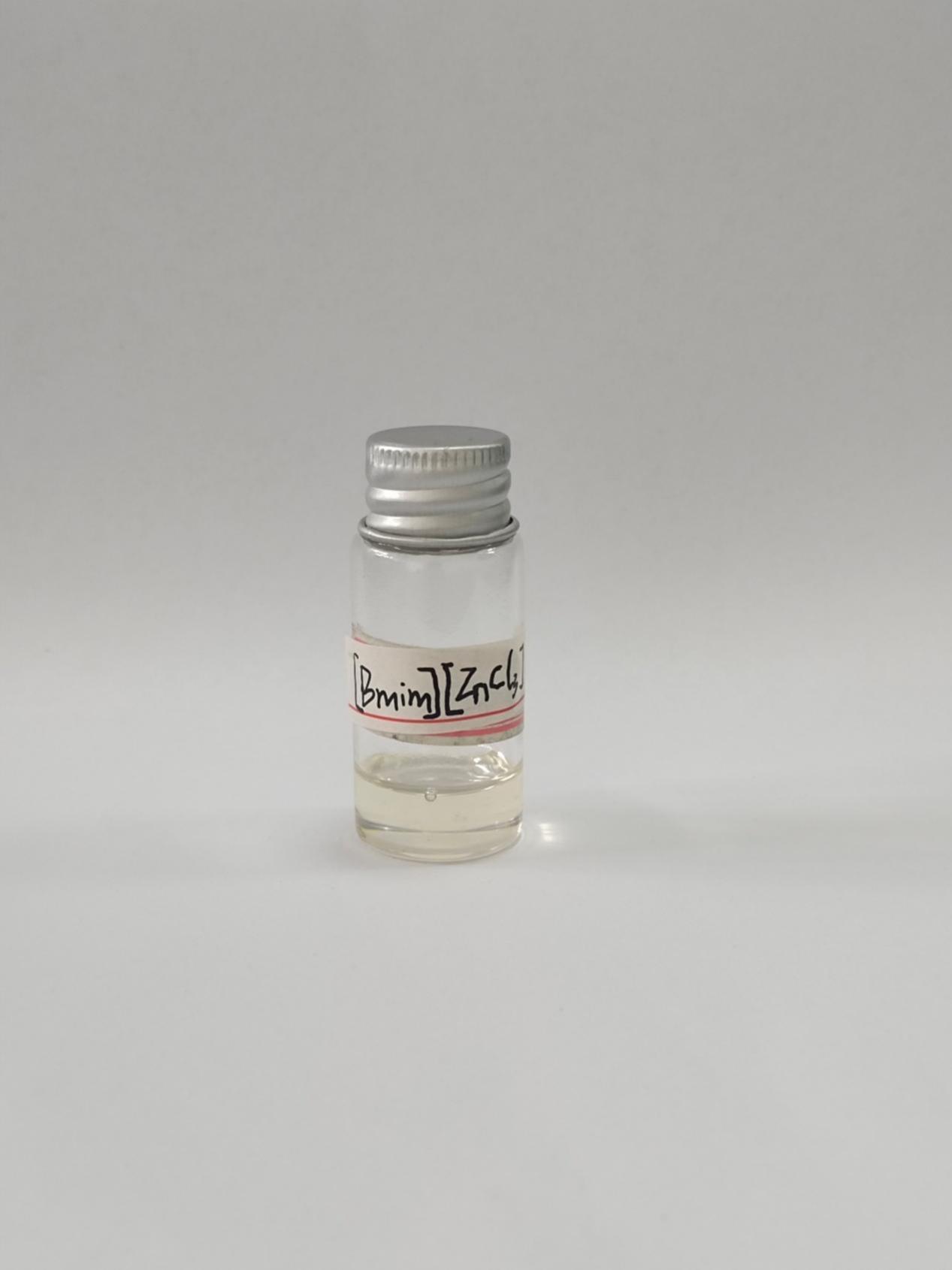


**Figure S1.** The photograph shows the macroscopic appearance of [Bmim][ZnCl_3_].

**Figure S2.** Photographs show the macroscopic appearance of a) [Bmim][ZnCl_3_]@ZIF-8-0%, b) [Bmim][ZnCl_3_]@ZIF-8-10%, c) [Bmim][CuCl_3_]@ZIF-8, d) [Bmim][CoCl_3_]@ZIF-8, e) [Bmim][MnCl_3_]@ZIF-8, and f) [Bmim][Cu/Co/MnCl_3_]@ZIF-8.

**Figure S3.** SEM images of a) [Bmim][ZnCl_3_]@ZIF-8-0%, b) [Bmim][ZnCl_3_]@ZIF-8-5%, c) [Bmim][ZnCl_3_]@ZIF-8-10%, and d) [Bmim][ZnCl_3_]@ZIF-8-15%.

**Figure S4.** a) and b) TEM images, c) HAADF-STEM image, and d) corresponding EDX elemental mapping images of [Bmim][ZnCl_3_]@ZIF-8-0%.

**Figure S5.** a) and b) TEM images, c) HAADF-STEM image, and d) corresponding EDX elemental mapping images of [Bmim][ZnCl_3_]@ZIF-8-10%.

**Figure S6.** XRD patterns of [Bmim][ZnCl_3_]@ZIF-8-0%, [Bmim][ZnCl_3_]@ZIF-8-5%, [Bmim][ZnCl_3_]@ZIF-8-10%, and [Bmim][ZnCl_3_]@ZIF-8-15%.

**Figure S7.** FTIR spectra of [Bmim][ZnCl_3_], [Bmim][ZnCl_3_]@ZIF-8-0%, and [Bmim][ZnCl_3_]@ZIF-8-10%.

**Figure S8.** a) N_2_ adsorption-desorption isotherms, b) pore-size distributions of [Bmim][ZnCl_3_]@ZIF-8-0%, [Bmim][ZnCl_3_]@ZIF-8-5%, [Bmim][ZnCl_3_]@ZIF-8-10%, and [Bmim][ZnCl_3_]@ZIF-8-15%.

**Figure S9.** XPS survey spectra of d-PNC(800, 10%), d-PNC(900, 10%), d-PNC(1000, 10%), and d-PNC(1100, 10%).

**Figure S10.** Variation of current versus concentration related cyclic voltammograms.

**Figure S11.** Effect of d-PNC(1100, 10%) on removing Cr(VI) in different small-molecule organic acid systems.

**Figure S12.** Effect of catalyst dosage on Cr(VI) reduction efficiency.

**Note for Figure S12:** Through systematic optimization of the d-PNC(1100, 10%) loading, we identified 0.06 g/L as the optimal catalyst dosage. This loading ensures that the catalytic activity is saturated while avoiding the unnecessary economic burden of excessive catalyst usage.

**Figure S13.** Effect of ratio of Cr(VI) to OA on Cr(VI) reduction efficiency.

**Note for Figure S13:** The dosage of OA is crucial for both the reduction efficiency and economic feasibility of Cr(VI) removal. Through systematic investigation of different [Cr(VI):OA] molar ratios (ranging from 1:2 to 1:10), we identified the optimal ratio of 1:7, at which the system achieved peak performance. Beyond this specific ratio, further increases in OA dosage yielded no significant enhancement in removal efficiency but substantially increased the operational cost.

**Figure S14.** SEM images of a) d-PNC(800, 10%), b) d-PNC(900, 10%), c) d-PNC(1000, 10%), and d) d-PNC(1100, 10%).

**Figure S15.** Model construction showing the interaction between OA and Cr(VI) in a) Defective C and b) Defective C + N4.

**Figure S16.** TGA-DTG curves of [Bmim][ZnCl_3_]@ZIF-8-0%, [Bmim][ZnCl_3_]@ZIF-8-5%, [Bmim][ZnCl_3_]@ZIF-8-10%, and [Bmim][ZnCl_3_]@ZIF-8-15% under N_2_ atmosphere.

**Figure S17.** SEM images of a) d-PNC(1100, 0%), b) d-PNC(1100, 5%), c) d-PNC(1100, 10%), and d) d-PNC(1100, 15%).

**Figure S18.** a) and b) TEM images, (b, inset) SAED pattern, c) HRTEM image with lattice distortions, d) HAADF-STEM image and corresponding EDX elemental mapping images of d-PNC(1100, 0%).

**Figure S19.** XPS survey spectra of d-PNC(1100, 0%), d-PNC(1100, 5%), d-PNC(1100, 10%), and d-PNC(1100, 15%).

**Figure S20.** a) and b) TEM images, c) SAED pattern, d) HRTEM image with lattice distortions, e) HAADF-STEM image and f) corresponding EDX elemental mapping images of used d-PNC(1100, 10%).

**Figure S21.** a) XPS survey spectra of d-PNC(1100, 10%) and used d-PNC(1100, 10%). b) high resolution XPS spectra in Cr 2p of used d-PNC(1100, 10%).

**Figure S22.** High resolution XPS spectra of a) C 1s, b) N 1s, c) O 1s of d-PNC(1100, 10%) and used d-PNC(1100, 10%).

**Figure S23.** UV-visible absorption spectra of OA, Cr(VI), a physical mixed solution of Cr(VI) with OA (without catalyst injection), solution after reaction of Cr(VI) with OA (with the addition of d-PNC(1100, 10%) catalyst), Cr(III) solution and a physical mixed solution of Cr(III) with OA.


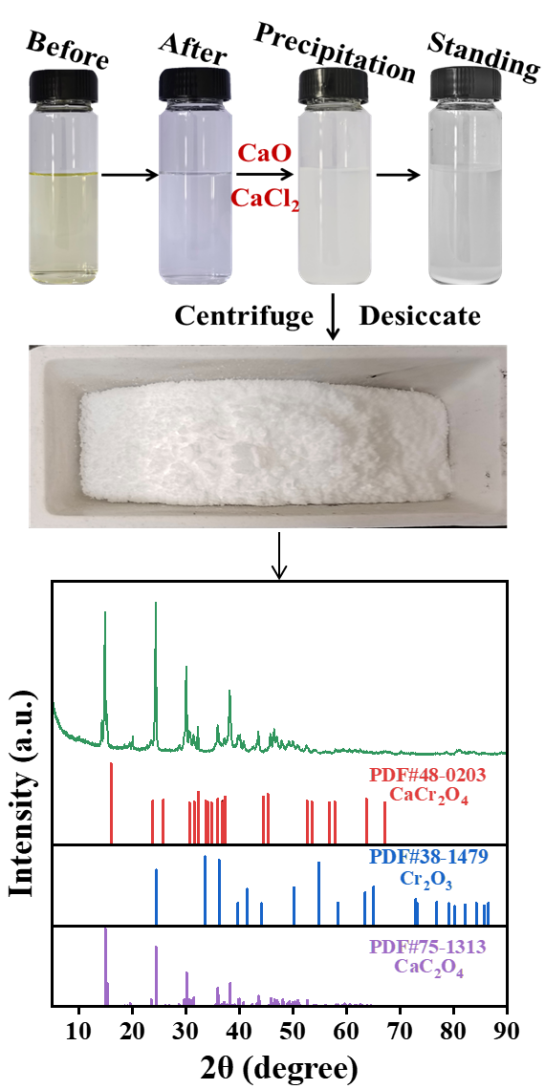


**Figure S24.** The complete process of Cr(VI) reduction is shown, including flow chart (top), sample picture of the reduced product after precipitation and drying (middle), and XRD pattern of the precipitate(bottom).

**Figure S25.** EPR spectra of d-PNC(1100, 10%)/OA/Cr(VI)/EHBA as the reaction progressed.

**Figure S26.** The electrochemical impedance spectroscopy of d-PNC(1100, 0%), d-PNC(1100, 5%), d-PNC(1100, 10%), and d-PNC(1100, 15%).

**Figure S27.** Photocurrent responses of d-PNC(1100, 0%) and d-PNC(1100, 10%).

**Figure S28.** The influence of reaction system composition on the reduction of U(VI). (Reaction parameters: catalyst dosage = 0.24 g·L^−1^, C_U(VI)_initial = 10 mg·L^−1^, C_OA_ = 0.24 g·L^−1^, room temperature, pH_initial_ = 2.5 without addition extra acid. No OA = d-PNC(1100, 10%) + U(VI), catalyst = d-PNC(1100, 10%) + U(VI) + OA).


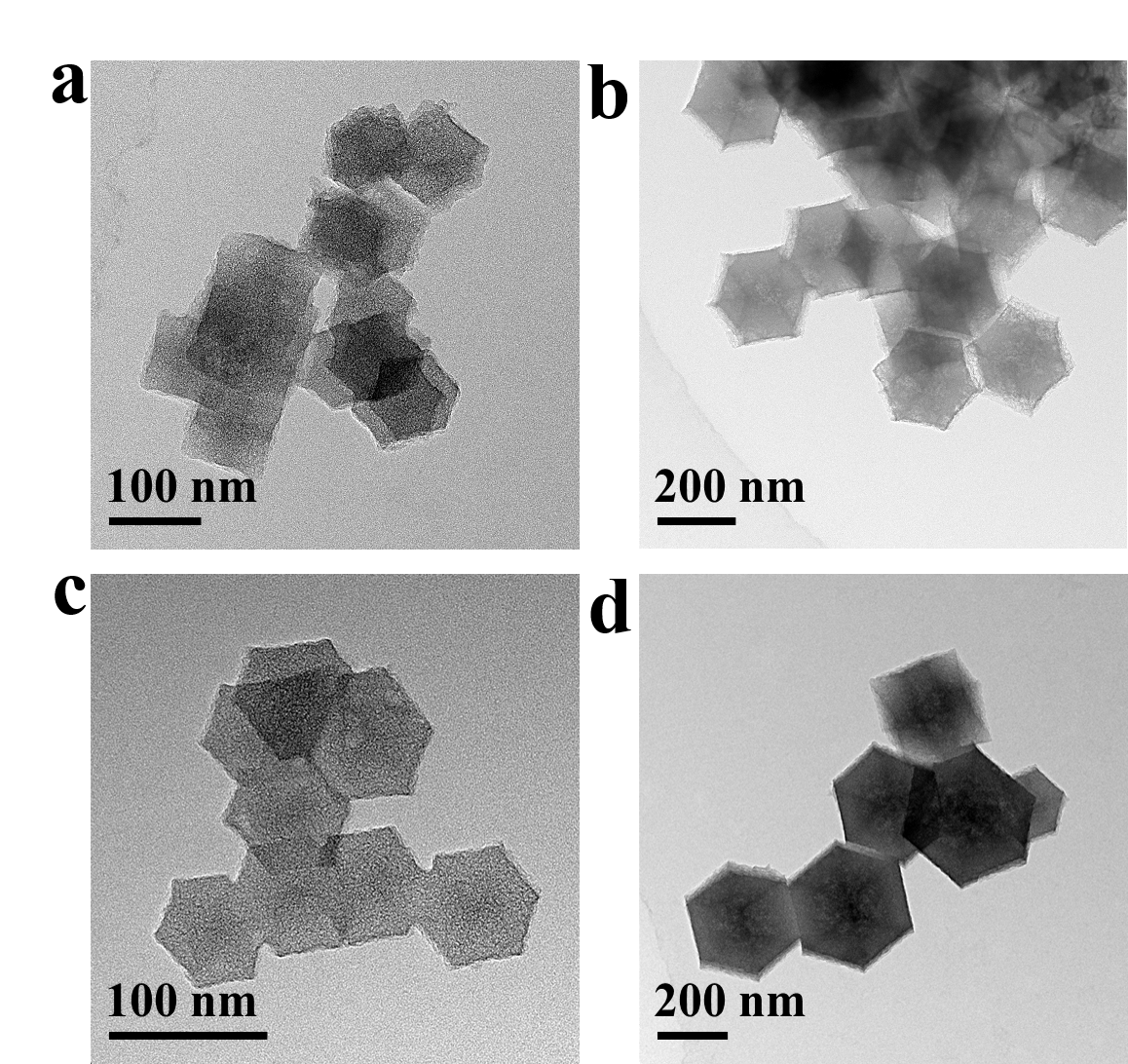


**Figure S29.** TEM images of a) Cu/d-PNC, b) Co/d-PNC, c) Mn/d-PNC, and d) Cu-Co-Mn/d-PNC.

**Figure S30.** Schematic diagrams of catalyst synthesis: a) Cu/d-PNC, b) Co/d-PNC, c) Mn/d-PNC, and d) Cu-Co-Mn/d-PNC.

**Figure S31.** Catalytic reduction Cr performance of d-PNC(1100,10%), Cu/d-PNC, Co/d-PNC, Mn/d-PNC, and Cu-Co-Mn/d-PNC.

**Table S1.** Physicochemical properties of catalyst.

| **Catalyst** | **BET Surface Area**  **(m^2^·g^−1^)** | **External**  **surface area**  **(m²·g^−1^)** | **Pore Volume**  **(cm^3^·g^−1^)** |
| --- | --- | --- | --- |
| d-PNC(800, 10%) | 716.90 | 110.61 | 0.39 |
| d-PNC(900, 10%) | 1160.93 | 126.02 | 0.57 |
| d-PNC(1000, 10%) | 1330.34 | 134.36 | 0.64 |
| d-PNC(1100, 10%) | 1636.90 | 230.43 | 0.83 |
| d-PNC(1100, 5%) | 1429.27 | 144.81 | 0.70 |
| d-PNC(1100, 15%) | 1326.30 | 180.59 | 0.71 |
| d-PNC(1100, 0%) | 1123.78 | 108.04 | 0.59 |
| [Bmim][ZnCl_3_]-ZIF-8-0% | 1958.91 | 40.61 | 0.77 |
| [Bmim][ZnCl_3_]-ZIF-8-5% | 1818.62 | 36.38 | 0.70 |
| [Bmim][ZnCl_3_]-ZIF-8-10% | 1795.38 | 33.11 | 0.66 |
| [Bmim][ZnCl_3_]-ZIF-8-15% | 1347.82 | 20.85 | 0.52 |

**Table S2.** The content of different N bonds in as-prepared d-PNC materials from high-resolution N 1s XPS spectra.

| **Catalysts** | **N1** | **N2** | **N3** | **N4** | **N5** |
| --- | --- | --- | --- | --- | --- |
| d-PNC(800, 10%) | 65.45 | 12.21 | 14.18 | 5.85 | 2.31 |
| d-PNC(900, 10%) | 44.31 | 12.08 | 31.19 | 8.80 | 3.62 |
| d-PNC(1000, 10%) | 33.30 | 9.80 | 37.77 | 10.90 | 8.13 |
| d-PNC(1100, 10%) | 25.13 | 8.43 | 42.80 | 14.25 | 9.40 |
| d-PNC(1100, 0%) | 23.96 | 10.13 | 42.60 | 13.26 | 10.55 |
| d-PNC(1100, 5%) | 20.29 | 10.83 | 46.30 | 12.78 | 9.80 |
| d-PNC(1100, 15%) | 24.12 | 8.13 | 45.20 | 12.69 | 9.87 |

**Table S3.** Comparison of catalytic performance for Cr(VI) reduction.

| Catalysts | Dosage (g·L^−1^) | C_Cr(VI)_ (mg·L^−1^) | n_Cr(VI)_/n_OA_ | Removal capacity (mg·g^−1^) | Time (min) | % | Ref. |
| --- | --- | --- | --- | --- | --- | --- | --- |
| Biochar-900-6 | 0.5 | 100 | 1:3.5 | 200 | 20 | 99.6 | [6] |
| Rice straw | 1 | 52 | 1:5 | 50 | 60 | 100 | [7] |
| PCNR-3-10-2-800 | 0.5 | 100 | 1:3.5 | 200 | 5 | 100 | [8] |
| S-nZVI | 0.3 | 52 | 1:6 | 121.1 | 120 | 70 | [9] |
| PLCN20 | 0.2 | 20 | 1:12.5 | 98.9 | 10 | 98.9 | [10] |
| Fe_3_O_4_@PANI (microwave) | 0.2 | 52 | 1:1 | 260 | 5 | 100 | [11] |
| 0.4% Fe-CN | 0.1 | 5 | 1:20.8 | 50 | 15 | 100 | [12] |
| Bio-Fe_2_O_3_/Fe_2_(WO_4_)_3_ | 0.3 | 20 | 1:5.2 | 66.7 | 60 | 100 | [13] |
| uPNC-NS-800 | 0.02 | 10 | 1:10 | 500 | 6 | 100 | [14] |
| d-PNC(1100, 10%) | 0.06 | 20 | 1:7 | 333.3 | 2 | 100 | **This work** |

**Table S4.** Reaction path energy of Defective C, Defective C + N3, and Defective C + N4.

|  | **Defective C** | **Defective C + N3** | **Defective C + N4** |
| --- | --- | --- | --- |
| H_2_C_2_O_7_Cr→*H_2_C_2_O_7_Cr | −2.438 | −1.933 | −1.630 |
| *H_2_C_2_O_7_Cr+(H^+^+e^−^) → *HC_2_O_6_Cr+H_2_O | −0.783 | −0.915 | −1.426 |
| *HC_2_O_6_Cr+(H^+^+e^−^) → *H_2_C_2_O_6_Cr | −0.085 | −0.120 | 0.007 |
| *H_2_C_2_O_6_Cr+(H^+^+e^−^) → *HC_2_O_5_Cr+H_2_O | 0.366 | 0.295 | 0.556 |

**Table S5.** The theoretical and experimental values of the intermediate and intermediate product charge ratio (m/z) detected chemical reduction of Cr(VI) by oxalic acid, as well as the relative error (***Δ***ppm).

| **Formula** | **Theoretical** | **Experimental** | ***Δ*ppm** | **Oxidation state** |
| --- | --- | --- | --- | --- |
| HCrO_4_^−^ | 116.92854 | 116.92908 | −4.62 | VI |
| HC_2_CrO_7_^−^ | 188.91329 | 188.91368 | −2.06 | VI |
| C_4_CrO_9_ | 243.89529 | 243.89575 | −1.88 | V |
| C_2_CrO_5_^−^ | 155.91563 | 155.91624 | −3.91 | III |
| C_4_CrO_8_^−^ | 227.90038 | 227.90140 | −4.48 | III |
| H_2_C_6_CrO_12_^−^ | 317.89569 | 317.89697 | −4.03 | III |

**Table S6.** EIS fitted parameters of d-PNC(1100, 0%), d-PNC(1100, 5%), d-PNC(1100, 10%), and d-PNC(1100, 15%).

| **Quantitative Parameters** | **Rs (Ω)** | **Rct (Ω)** |
| --- | --- | --- |
| d-PNC(1100, 0%) | 2.993 | 209.4 |
| d-PNC(1100, 5%) | 5.376 | 151.5 |
| d-PNC(1100, 10%) | 5.485 | 124.1 |
| d-PNC(1100, 15%) | 2.792 | 133.4 |

**Table S7.** The conventional water quality indexes of pH, common anions, and cations.

| ^c^Actual water sample | ^a^Anion (mg/L) | | | ^b^Cation (mg/L) | | | | | | pH |
| --- | --- | --- | --- | --- | --- | --- | --- | --- | --- | --- |
|  | NO_3_^-^ | SO_4_^2^ | Cl^-^ | Na^+^ | K^+^ | Ca^2+^ | Cr^6+^ /Cr^3+^ | Fe^3+^ /Fe^2+^ | Zn^2+^ |  |
| Ultrapure water | <0.1 | <0.1 | <0.1 | <0.1 | <0.1 | <0.1 | <0.1 | <0.1 | <0.1 | 7.7 |
| Tap water | 3.3 | 14.6 | 6.1 | 42.7 | 24.9 | <0.1 | <0.1 | <0.1 | <0.1 | 7.0 |
| Lake water | <0.5 | 809.3 | 618.8 | 4.1 | 4.4 | 20.4 | <0.1 | <0.1 | <0.1 | 6.5 |
| Sea water | <0.5 | 9.1×10^4^ | 5.8×10^6^ | 3.0×10^3^ | 301.9 | 164.5 | <0.1 | <0.1 | <0.1 | 6.3 |
| Mineral water | <0.1 | <0.1 | <0.1 | <0.1 | <0.1 | <0.1 | <0.1 | <0.1 | <0.1 | 7.8 |
| Electroplating  wastewater | 749.9 | 18.0 | 1921 | 30.9 | 374.2 | 0.3 | 107.7 | <0.1 | <0.1 | 11.3 |

^a^Detected by IC.

^b^Detected by ICP-OES.

^c^The origins of the water samples used in the study were as follows:

(1) Tap water was sourced from the Dalingshan town in Dongguan city.

(2) Lake water was obtained from Songshan Lake in Dongguan city.

(3) Sea water was collected from Shenzhen Bay.

(4) Mineral water refers to Nongfu Spring mineral water.

(5) River water originates from the Pearl River in Guangzhou city.

(6) Electroplating wastewater was obtained from Anke Hardware & Plastic Surface Treatment Co., Ltd in Huizhou city.

**References**

[1] G. Kresse, J. Furthmüller, *Computational Mater. Sci.* **1996**, *6*, 15.

[2] G. Kresse, J. Hafner, *Phys. Rev. B* **199**4, *49*, 14251.

[3] J. P. Perdew, K. Burke, M. Ernzerhof, *Phys. Rev. Lett.* **1996**, *77*, 3865.

[4] G. Kresse, D. Joubert, *Phys. Rev. B* **1999**, *59*, 1758.

[5] P. E. Blöchl, O. Jepsen, O. K. Andersen, *Phys. Rev. B* **1994**, *49*, 16223.

[6] D. Yang, T. Liu, R. Deng, Z. Xian, Y. Chen, *Chem. Eng. J.* **2024**, *499*, 156556.

[7] L. Zhang, J. Sun, W. Niu, F. Cao, *Environ. Pollut.* **2020**, *265*, 115013.

[8] D. Yang, R. Deng, M. Chen, T. Liu, L. Luo, Q. He, Y. Chen, *J. Hazard. Mater.* **2023**, *459*, 132283.

[9] Y. Yuan, X. Wei, H. Yin, M. Zhu, H. Luo, Z. Dang, *J. Hazard. Mater.* **2022**, *423*, 127240.

[10] M. Li, H. Dai, P. Zhan, C. Tan, Z. Ning, F. Hu, X. Xu, X. Peng, *J. Clean. Prod.* **2023**, *415*, 137883.

[11] C. Zhu, F. Liu, L. Song, H. Jiang, A. Li, *Environ. Sci.: Nano* **2018**, *5*, 487.

[12] X. Zhang, J. Liu, X. Zheng, R. Chen, M. Zhang, Z. Liu, Z. Wang, J. Li, *Appl. Catal. B Environ. Energy* **2023**, *321*, 122068.

[13] C. Gu, Q. Yang, X. Zhang, R. Feng, S. Wang, T. Liu, P. He, H. Yin, J. Zhu, M. Gan, *Water Res*. **2025**, *283*, 123832.

[14] D. Lin, X. Lin, H. Li, H. Huang, H. Yang, J. Yang, B. Hu, F. Chen, X. Guo, X. Wang, W. Yao, *Appl. Catal. B Environ. Energy* **2025**, *370*, 125174.
